# Nanocomposites of organo-montmorillonite/polystyrene latex particles *via* free radical miniemulsion polymerization†

**DOI:** 10.1039/d4ra08943j

**Published:** 2025-02-18

**Authors:** Ahmed Akelah, Ahmed Rehab, Hisham Harhash, Mohamed A. Abdelwahab, Hamada S. A. Mandour

**Affiliations:** a Polymer Research Group, Chemistry Department, Faculty of Science, Tanta University Tanta 31527 Egypt mohamed.abdelwahab@science.tanta.edu.eg

## Abstract

Encapsulation of high-aspect-ratio inorganic particles by polymeric materials is still a challenge; armored or dumbbell-like morphologies were frequently the outcome. In this paper, through miniemulsion polymerization, organo-montmorillonite (MMT) platelets have been successfully encapsulated by polystyrene (PS). First, MMT was hydrophobized by cation exchange with a polymerizable interlayer spacer (*N*-allyl-*N*,*N*-dimethyloctadecan-1-aminium bromide), which not only widened the interplanar spacing but also facilitated monomer intercalation into the MMT nanogalleries and provided a covalent anchor with PS chains. Then, the swelled modified MMT in the monomer phase was combined with a surfactant, costabilizer, and initiator to obtain stable nanocomposite latexes. Analytical techniques, including FTIR, XRD, SEM, TEM, and TGA, provided comprehensive insights into the structural and thermal properties of these nanocomposites. XRD analyses unveiled an exfoliated structure. Furthermore, the TEM micrographs showed the formation of stable spherical particles with diameters ranging from 250 to 465 nm containing encapsulated MMT. These nanocomposites exhibited significant improvement in thermal stability—decomposing at elevated temperatures compared to virgin PS. This work opens new avenues for encapsulating other high-aspect-ratio nanofillers in a diverse array of polymer types, leading to the synthesis of innovative materials with enhanced properties for broader practical applications. Furthermore, it can be used for controlled-release applications, particularly in drug delivery and agriculture.

## Abstract 2

Hydrophobization of MMT with a reactive ADM_18_ molecule and its encapsulation into polystyrene latex particles *via* miniemulsion polymerization.

## Graphical abstract

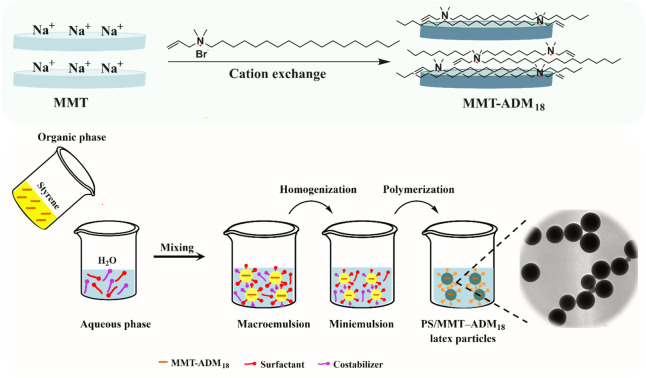

## 1. Introduction

Nanoscale lamellar silicate-reinforced polymeric nanocomposites have garnered significant interest worldwide in both academic and industrial research because of their outstanding features and growing range of applications.^1–3^ Because these materials effectively take advantage of the synergy between a polymer matrix and nanoplatelet fillers, even at low or moderate (1–5%) reinforcing filler loading, they exhibit improved mechanical and thermal properties, reduced gas permeability, and decreased flammability when compared to virgin polymers.^4–6^ The advantages are obtained with full delamination and a random distribution of clay layers. These improvements stem from robust interfacial interactions between the polymer matrix and the individual silicate platelets.^2,4^ According to the interphase force between the layered silicate and the polymeric matrix, the resultant nanocomposite is categorized into intercalated nanocomposite; as a consequence, a finite expansion of interlayer spacing is produced when the layers of clay are separated by polymer chains arranged in a regular crystallographic manner. In contrast, the substantial penetration of the polymer chains into the interlayer space led to exfoliation, which is the whole or partial delamination of individual layers throughout the polymer matrix.^1,7,8^

Most published work on polymer-clay nanocomposites (PCNs) has focused on MMT, an aluminosilicate smectite clay. MMT is a layered, hydrated aluminum phyllosilicate with a plate-like shape. It consists of two sheets of silicate/aluminum oxide sandwiched between an edge-shared octahedral sheet and a corner-linked tetrahedral structure.^9,10^ To successfully produce PCNs, natural clay's hydrophilic properties must be altered. This is because the inherent hydrophilicity and strong interlayer interaction of pristine clay nanoparticles cause them to be incompatible, show weak interfacial interactions, and display poor dispersion with or in most organic polymeric materials. One of the most prevalent methods to prepare organoclay is the ion-exchange reaction between the stabilizing cation within layered structures and organic onium ions that contain a long hydrophobic chain, which not only enlarges the gallery space but also bears a reactive end group that is capable of covalently bonding to the monomer, triggering clay exfoliation within the polymer matrix and thus improving the technical features of the nanocomposite.^2,11,12^

Various techniques have been employed to create PCNs, the most common being *in situ* polymerization. Melt processing and solution mixing techniques have also been employed on occasion.^7,8,13^*In situ* polymerization requires the pre-dispersion of organoclay in the liquid monomer or solution of monomer, which facilitates better diffusion of the monomer molecules into their galleries, and then inter-gallery polymerization occurs. Owing to the many distinct polymerization conditions and clay treatments that can be employed for obtaining exfoliated morphology, *in situ* polymerization is the approach of choice. Out of all the *in situ* polymerization methods, water-borne *in situ* polymerization is the most appealing, as it is easier to implement and more eco-friendly than traditional solvent-borne approaches for the production of PCNs.^7^ There are several dispersed-phase polymerization processes for synthesizing polymeric nanocomposites. The most well-known method is emulsion polymerization. Emulsion polymerization is a heterophase polymerization that spreads the monomer into droplets roughly 10 μm in diameter. The surfactant's adsorption at the monomer–water interface is liable for droplet stability. Surfactant forms micelles if its concentration exceeds the critical micelle concentration (CMC). Micelles are swollen by absorbing monomer molecules. Due to their small size (10 nm in diameter), monomer-swollen micelles are the main loci of nucleation, so the optimal mechanism is micellar nucleation. Polymerization starts with the reaction of water-borne radicals with monomer molecules dissolved in the aqueous phase, forming oligomeric radicals. They grow up in the aqueous phase until they become so hydrophobic to enter the monomer-swollen micelles and react with the monomer within to cause chain propagation. Monomer droplets supply the growing particles with monomers through the water. The product is a dispersion of small polymer particles stabilized by the surfactant molecules (latex). The process of emulsion polymerization includes three main intervals. Interval I is the period during which particles are nucleated. Micelles are the main polymerization loci. With time, the number of particle nuclei and the polymerization rate increase. In interval II, no new particles are nucleated, and the growing polymer particles are continuously fed with the monomer from monomer droplets. During this interval, the number of particles as well as the polymerization rate remain constant. Monomer droplets progressively decrease until they disappear at the end of interval II. In interval III, all monomer droplets disappear, and the remaining monomer contained within the particles undergoes polymerization. The rate of polymerization decreases progressively as the remaining monomer is consumed. The final product is the emulsion of polymer in water called latex.^14,15^ Nanocomposite polymer latexes can also be produced through miniemulsion polymerization. Using this method, synthesized polymeric or inorganic materials may be well-encapsulated.^16^

To produce a somewhat stable submicron dispersion of oil in water, miniemulsions are often made by applying a high-shear system that consists of a monomer, water, surfactant, and costabilizer. The combination of an effective surfactant and costabilizer prevents droplet collisions and diffusional degradation, giving droplets stability.^17,18^ The nanosized monomer droplets that formed by applying high shear to the system have a sufficiently large surface area to effectively compete with the micelles or particles for radical capture. To stabilize the large droplet surface area, more surfactant from the aqueous phase is adsorbed. This leads to little or no surfactant in the aqueous phase, which prevents the production of particles by micellar or homogenous nucleation. Consequently, droplet nucleation becomes the prime mechanism. Particles are generated by hitting monomer droplets with primary or oligomeric radicals, and then reacting with the monomers therein. In contrast to homogeneous or micellar nucleation, oligomeric radicals nucleate nearly all monomer droplets and transform them into latex particles. Latex particle droplets are frequently one-to-one replicas of the original droplets, which is explained by the reality that the droplets serve as the locus of nucleation.^14,17^

Miniemulsion polymerization has shown effectiveness in encapsulating a variety of spherical inorganic particles, such as silica,^19,20^ magnetite,^21^ titanium dioxide,^22^ and silver particles.^23^ However, encasing high-aspect-ratio inorganic particles presents further challenges due to their ability to form stacks and card-house structures. Modified Laponite,^24^ Saponite,^25^ or MMT^26^ were present in almost all instances. There are widely challenges regarding encapsulating MMT in polymer particles utilizing emulsion-based polymerization; most of the time, clay-armored particles are the result.^27^ Only a few studies have attempted to encapsulate MMT platelets inside latex particles *via* emulsion or miniemulsion polymerization. For instance, Mirzataheri *et al.*^28^ prepared poly(styrene-*co*-butyl acrylate)/cloisite (30B) nanocomposites *via* miniemulsion polymerization. Their findings showed that the nanocomposites were fully exfoliated. Also, the TEM image revealed the formation of spherical particles containing cloisite 30B within the polymer latex. The difficulty in identifying MMT platelets from the TEM images gives rise to concerns surrounding true encapsulation. Voorn *et al.*^27^ presented a powerful example of partial encapsulation of MMT by polymerizing reactive silane-modified MMT with PMMA using emulsion polymerization. According to TEM findings, a dumbbell-like morphology containing MMT was observed. The high viscosity of the organic phase has mainly restricted the percentage of encapsulated clay to 4%. Consequently, the MMT cannot be finely distributed, which will eventually lead to colloidal instability.^25,29^

This work focuses on synthesizing and characterizing nonpolar PS-encapsulated nanoscale MMT platelets generated through the miniemulsion polymerization technique. MMT was modified using polymerizable ammonium salt, which could copolymerize with nonpolar styrene monomer, causing exfoliation and facilitating encapsulation. The newly synthesized nanocomposites will be morphologically and thermally characterized.

## 2. Experimental

### 2.1. Materials

Sodium montmorillonite (Na-MMT) was supplied from ECC America Inc. (USA), with a CEC of 90 mequiv per 100 g of clay. According to the previous study,^30,31^*N*-allyl-*N*,*N*-dimethyloctadecan-1-aminium bromide (ADM_18_) was synthesized and characterized by ^1^H NMR and mass spectroscopy. Styrene (St, stabilized with 4-TBC) was purchased from Sigma-Aldrich (Darmstadt, Germany). The inhibitor was removed after washing with a 1 M NaOH solution and distilled water and drying over anhydrous Na_2_SO_4_. Sodium dodecyl sulphate (SDS) was obtained from Techno Pharmchem (Bahadurgarh, India). Cetyl alcohol (CA) from Qualikems (Gujarat, India). Potassium persulfate (KPS) was provided by Fisher Scientific (USA). Sodium hydrogen carbonate was utilized as a buffer.

### 2.2. Preparation of materials

#### 2.2.1. Synthesis of organo-cationic modified MMT (MMT-ADM_18_)

The cation–exchange reaction was performed by swelling 3 g of MMT overnight in 90 mL of distilled water. 10 mL of dioxane was added to the swollen MMT with stirring. After heating 1.64 g of ADM_18_ in 30 mL of dioxane for one hour at 60 °C, it was gradually added to the swollen MMT while stirring at the same temperature. An abundant fine white precipitate was obtained after stirring for 5 h at 60 °C. After cooling, the obtained white precipitate was filtered and underwent several hot washes with 90/10 (v/v) water/dioxane to remove excess ADM_18_ molecules. Finally, the precipitate MMT-ADM_18_ was recovered and vacuum-dried at 30 °C.^10,32^

#### 2.2.2. Miniemulsion polymerization of styrene in the presence of MMT-ADM_18_

Table 1 shows the procedures to prepare the miniemulsion's monomer and aqueous phases. In the monomer phase, MMT-ADM_18_ (1–5% based on monomer content) was swelled in styrene monomer, then magnetically stirred and purged with N_2_ overnight at room temperature. To ensure complete swelling of the organo-MMT in monomer, the mixture was sonicated for 10 min under cooling. For 1 h at 65 °C, the aqueous phase, composed of distilled water (30 mL), cetyl alcohol (CA, 0.265 g), and sodium dodecyl sulphate (SDS, 0.105 g), was magnetically stirred to form a homogenous solution. The gel phase is broken up by sonication after the mixture has reached room temperature. Stable emulsions were produced with a molar ratio of 1 : 3 for SDS to CA.^17,33–35^ In a bath of ice, both phases were combined vigorously for 30 min, followed by rotor–stator homogenization for 10 min. The latexes are batch-wise synthesized; therefore, the resulting milk miniemulsion was poured into a three-neck flask containing (50 mg of NaHCO_3_ in 5 mL of distilled water), fitted with a condenser and a mechanical stirrer. Under nitrogen protection, the miniemulsion was agitated mechanically for 30 min. After the temperature stabilized at 72 °C, the KPS solution was added, and the polymerization was then left to continue for a whole night at 72 °C. After being cooled, the emulsion was coagulated with methanol to obtain the PS/MMT-ADM_18_ samples, which were then washed with distilled water. The white powder product was finally obtained after drying at 30 °C under vacuum. The nanocomposites prepared with 1, 3 and 5% MMT-ADM_18_ loadings were denoted as PS–1% MMT-ADM_18_, PS–3% MMT-ADM_18_, and PS–5% MMT-ADM_18_, respectively. Blank miniemulsion was prepared using the previously mentioned procedure for comparative purposes, with monomer conversion close to 96%.

**Table 1:** The recipe for forming PS/MMT-ADM_18_ nanocomposites *via* miniemulsion polymerization^a^

Phases	Components	Weight and concentration
Monomer phase	Styrene	5 g
	MMT-ADM_18_	0.05–0.25 g
Aqueous phase	Water	45 mL
	SDS	0.105 g (10 mM)
	CA	0.265 g (30 mM)
	KPS	0.150 g (12 mM)
	NaHCO_3_	Same as KPS

a In parentheses, relative to the aqueous phase.

### 2.3. Analytical procedures and measurements

Fourier transform infrared spectrometry (FTIR) spectra were recorded in the 4000–400 cm^−1^ range by a JASCO FTIR-4100 spectrometer (Japan) using the KBr disk technique.

X-ray diffraction (XRD) analysis was performed on a Phillips X-ray diffractometer (PW-1710) using a Cu-Ka radiation source (*λ* = 0.154060 nm). The scan was from 3° to 80° (2*θ*) at 3° min^−1^ scan speed.

Thermogravimetric analysis (TGA) was conducted using a TGA-50 Shimadzu analyzer (Shimadzu Corp, Kyoto, Japan). 10 mg of each sample was heated to 800 °C in an air atmosphere at a rate of 10 °C min^−1^.

The average particle size and particle size distribution were measured using dynamic light scattering (DLS) on a Zetasizer Nano ZS from Malvern Instruments Ltd (UK).

Scanning electron microscopy (SEM) and transmission electron microscopy (TEM) were employed to examine the nanomorphology of the composites. SEM was conducted on a JEOL JSM-IT200 (JEOL, Tokyo, Japan) operating at an accelerating voltage of 20 kV. Energy dispersive X-ray (EDX) analysis was installed with an SEM microscope. TEM images were acquired using an accelerating voltage of 120 kV on a JEOL JEM-1400 Plus (JEOL, Tokyo, Japan). In brief, a PS/MMT-ADM_18_ sample suspended in the acetone was sonicated, then dropped on a copper grid, and then the drops were evaporated.

## 3. Results and discussion

### 3.1. Characterization of MMT-ADM_18_

To improve MMT compatibility with the oil phase and facilitate encapsulation into monomer droplets upon emulsification, MMT was modified with *N*-allyl-*N*,*N*-dimethyloctadecan-1-aminium bromide (ADM_18_) to strengthen interaction with hydrophobic monomers and facilitate encapsulation during the emulsification process. After the cation exchange reaction, the structure of the resulting MMT-ADM_18_ was characterized by FTIR, XRD, and TGA analyses.

**Fig. 1:**
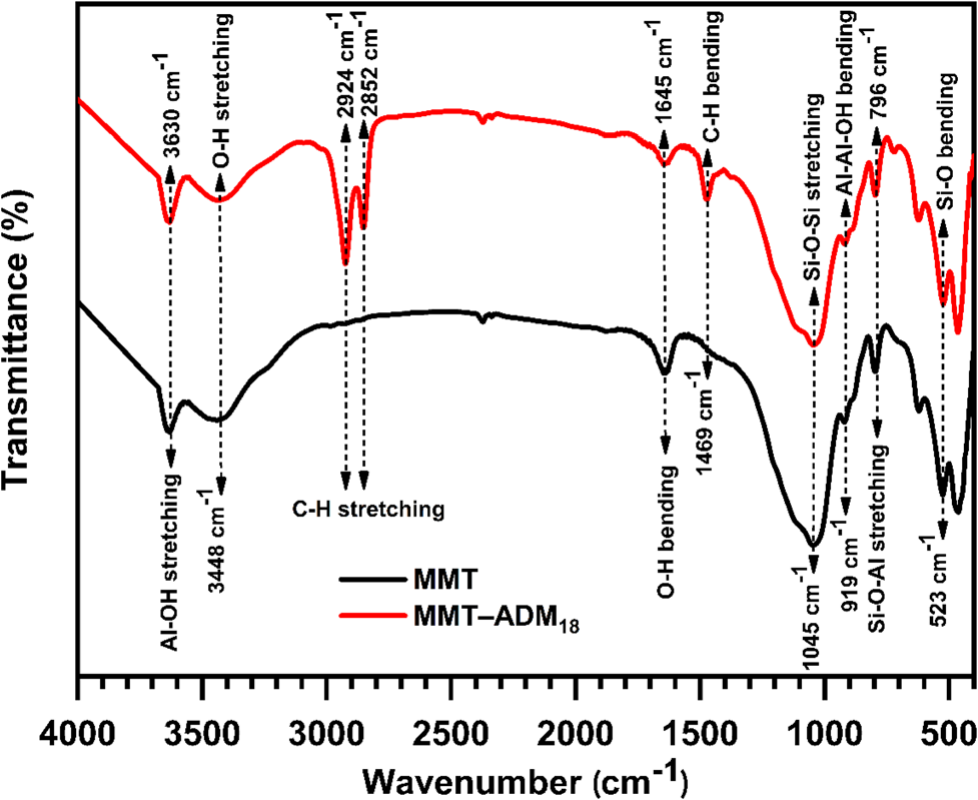
FTIR spectra of MMT and MMT-ADM_18_.

Fig. 1 shows the FTIR spectra of MMT and MMT-ADM_18_. MMT displays characteristic bands at 3630 cm^−1^ and 3448 cm^−1^, attributed to the (Al, Mg)–OH stretching vibrations and –OH stretching vibrations of H_2_O existed in the MMT interlamellar, respectively. Moreover, MMT displays a band at 1645 cm^−1^, which was attributed to the interlayer water molecules' OH bending vibration. The strong absorption peak at 1045 cm^−1^ belongs to the stretching vibration of Si–O–Si. The peak at 919 cm^−1^ was assigned to the Al_2_OH bending vibration, while the peak value at 796 cm^−1^ was attributed to the Si–O–Al stretching vibrations. The bending vibrations peak of Si–O and Al–O occurred at 600–400 cm^−1^ region.^4^ In contrast, for the MMT-ADM_18_ spectrum, the characteristic bands at 2924 and 2852 cm^−1^ are attributed to C–H stretching in the ammonium salt's alkyl chain, while the band at 1469 cm^−1^ is due to the C–H bending vibration of the alkyl chain.

**Fig. 2:**
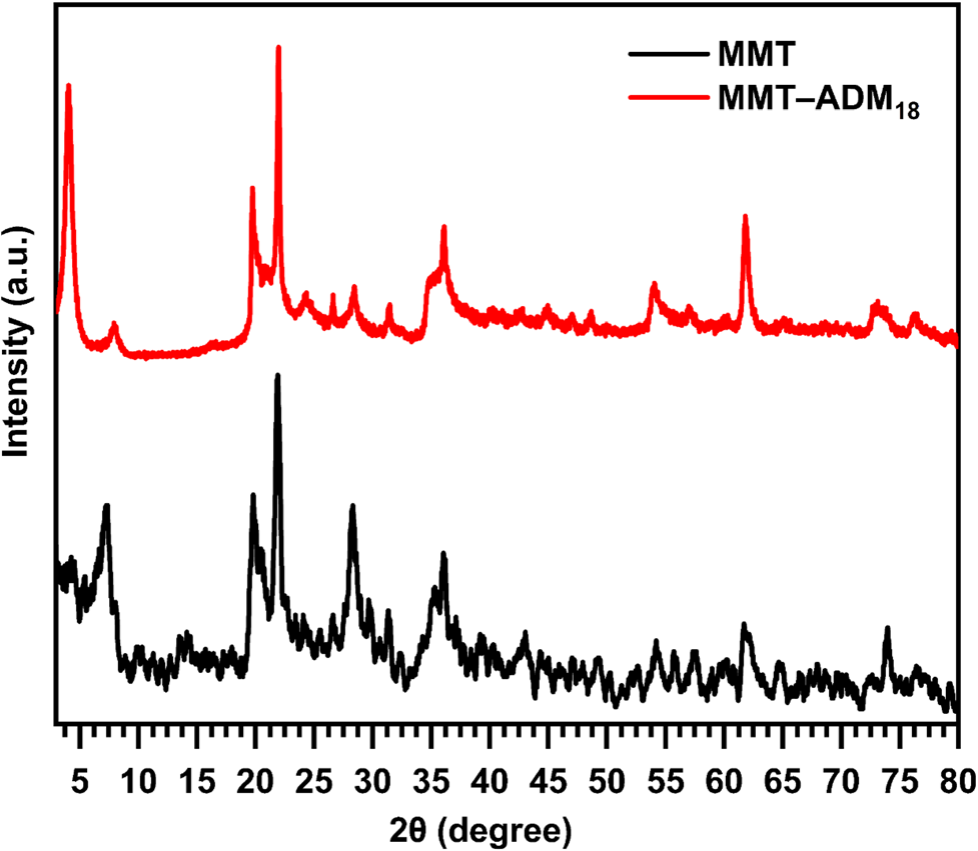
XRD patterns of MMT and ADM_18_-modified MMT.

XRD analysis is used to study the silicate gallery structure. Fig. 2 displays the XRD patterns of MMT and MMT-ADM_18_. The MMT characteristic diffraction peak appeared at 2*θ* = 7.18° with an interlayer spacing of 1.22 nm. After modification with quaternary ammonium salt, the diffraction angle decreases to 4° (2*θ*), which means that the spacing between the basal plane has increased to 2.18 nm. The increase in interlamellar space is due to larger ADM_18_ ions successfully replacing smaller Na^+^ ions in MMT lamellar galleries.^10^

**Fig. 3:**
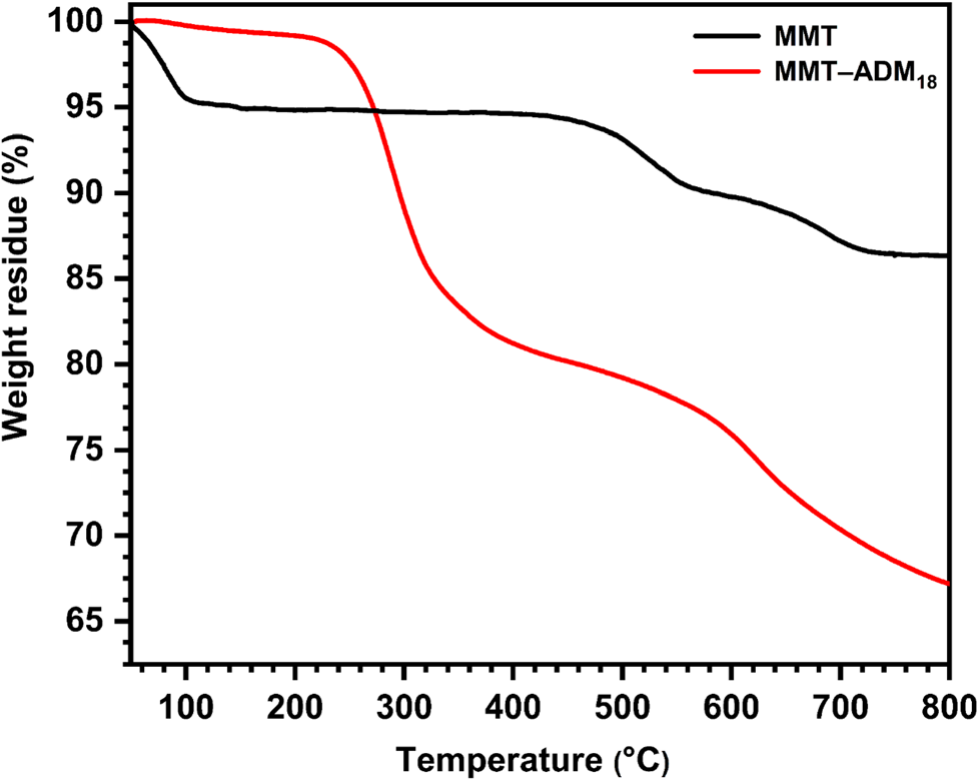
TGA profiles of MMT and MMT-ADM_18_.

The thermal stability of MMT and MMT-ADM_18_ provided additional evidence for the intercalation. As shown in Fig. 3, MMT lost around 4% when the temperature was below 100 °C because of the loss of surface and interlayer water molecules. MMT showed a degradation between 500 and 800 °C (around 6.5% loss of its weight) due to dehydroxylation of the layer crystal lattice structure of the MMT. MMT's overall loss was about 10.5%.^36^ In contrast, MMT-ADM_18_ exhibits a slight drop in weight below 100 °C due to less interlayer water as the sodium cations have been exchanged for ammonium ions. It was found that MMT-ADM_18_ began to degrade at ∼230 °C, to the clay's edges losing their quaternary ammonium cation anchorage.^37^ A 9% drop in weight was observed until the degradation extended to 290 °C. A further stage of degradation proceeded between 300 and 500 °C, causing 18% loss in weight, attributed to the decomposition of interlayer ADM_18_ cations. Over 600 °C, continued weight loss because the structural water in the MMT layers was removed. Overall, MMT-ADM_18_ lost 33% of its weight. Based on TGA analysis utilizing equations,^38^ the amount of intercalated ADM_18_ was 82.7 mequiv/100 g (91% of exchangeable sites). Also, elemental analysis confirmed the amount of ADM_18_ intercalated. The DLS plot (ESI Fig. S1†) for MMT-ADM_18_ shows that the average particle diameter is 695 nm with a narrow size distribution. This indicates the successful modification of MMT by ADM_18_ ions, which in turn enhanced the compatibility of MMT-ADM_18_ with nonpolar styrene and improved its dispersion in the matrix. Therefore, the results summarized in Table 2 confirm that the ADM_18_ ions successfully intercalated in MMT galleries.

**Table 2:** Summary of organic content, elemental analysis of MMT-ADM_18_, and the corresponding amount of intercalated ADM_18_

Sample	Organic fraction^a^ (%)	Organic fraction^b^ (%)	Intercalated ADM_18_^c^ (mequiv g^−1^)	CEC^c^ (%)	Z-average^d^ (nm)
MMT-ADM_18_	32.88	27.82	0.827	91	695

a Estimated from TGA.

b Estimated from elemental analysis.

c Intercalated ADM_18_ expressed in mequiv g^−1^ or percentage.

d Determined by DLS.

### 3.2. Miniemulsion polymerization

A high-shear emulsification process yields a stable submicron oil dispersion in water, known as a miniemulsion. During polymerization, the surfactant/costabilizer system preserves the droplets' stability. Ionic surfactants (*e.g.*, SDS) are amphiphilic compounds bearing charged end groups. They prevent droplet collisions by forming a charged shell around the droplets, resulting in electrostatic repulsion between them.^39^ In miniemulsions, the most common destabilizing mechanism is Ostwald's ripening. It occurs due to the difference in chemical potential in the variously sized droplets. The driving factor for transferring mass from the smaller to the bigger droplets is Laplace pressure, which causes chemical potential in smaller droplets to be greater than in bigger ones. As a result, bigger droplets grow and smaller ones shrink. When a costabilizer substance is added to the dispersed stage, it opposes the droplet's Laplace pressure and thereby reduces the process of ripening. The agent must be selected in such a way that it is confined within each droplet and is unable to disperse between them. Because the chemical potentials of the droplets vary, monomers can migrate from the small droplets to the big ones through the water phase. The monomers' concentration varies between the big and tiny droplets as a result. Due to their restricted affinity for water, the hydrophobe molecules are unable to diffuse out of the droplets; as a result, their concentration will increase in the small droplets (osmotic pressure will rise) and decrease in the large ones (osmotic pressure will decrease). Consequently, the monomer is compelled to move back to the tiny droplet, resulting in the formation of a stable miniemulsion, as the osmotic pressure opposes the forces of Laplace pressure.^40,41^ A more stable emulsion is produced by using a combined system containing SDS and CA. With water, CA forms a hydrogen bond thanks to its hydroxy end group. Consequently, the interspace between SDS molecules close to the droplet surface layer allows close packing for CA. Thus, the resulting intermolecular complex renders the miniemulsion intensely resistant to droplet aggregation as well as low interfacial tension. Additionally, the presence of CA reduces the monomer's droplet size, which increases the monomer droplets' ability to capture radicals for two reasons: (i) increasing the monomer droplets' surface area, and (ii) because of the increased surface area, more surfactant is adsorbed on the droplet's surface. Hence the free-surfactant present in the aqueous phase decreased (micellar nucleation minimized), causing droplet nucleation to be the predominant mechanism. Also, CA functions as a hydrophobic portion to prevent Ostwald ripening (monomer diffusion from small to large droplets), keeping the droplets stable for extended periods (weeks to months).^34,42^ With miniemulsion polymerization, however, every droplet may function as an independent nanoreactor, facilitating the reinforcing filler's uniform distribution throughout the polymer particles. Therefore, this polymerization technique is a promising route to encapsulate organo-modified MMT into the polymer particles.

### 3.3. Characterization of colloidal PS/MMT-ADM_18_ nanocomposites

#### 3.3.1. FTIR characterization

**Fig. 4:**
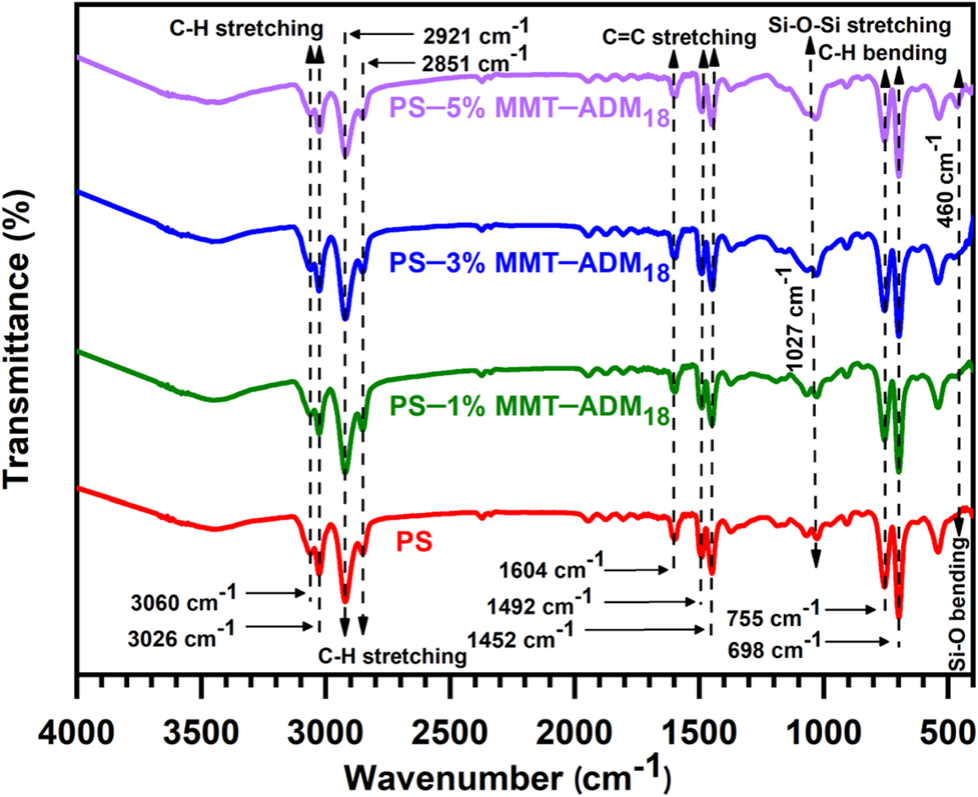
FTIR spectra of PS and PS/MMT-ADM_18_ nanocomposites with 1, 3, and 5% MMT-ADM_18_.

Fig. 4 displays the FTIR spectra of neat PS and PS nanocomposites with 1, 3, and 5% MMT-ADM_18_. PS showed characteristic bands at 3060 and 3026 cm^−1^, corresponding to aromatic C–H stretching. Peaks at 2921 and 2851 cm^−1^ correspond to CH_2_ asymmetric and symmetric stretching, respectively. The peaks at 1604, 1492 and 1452 cm^−1^ represent the benzene ring and C=C's stretching vibrations, and those at 755 and 698 cm^−1^ represent the aromatic ring's deformation vibrations.^5,43^ In the FTIR spectra of PS/MMT-ADM_18_ nanocomposites (1–5%), the characteristic peaks that appeared in the PS spectrum (C–H and C=C stretching vibrations) were visible. Moreover, a new peak that appeared in the regions of 1027 and 460 cm^−1^, corresponding to Si–O stretching and bending vibrations of MMT-ADM_18_. According to the above spectral analysis and comparison, it is certain that the PS/MMT-ADM_18_ nanocomposites have been synthesized successfully.

#### 3.3.2. XRD characterization

**Fig. 5:**
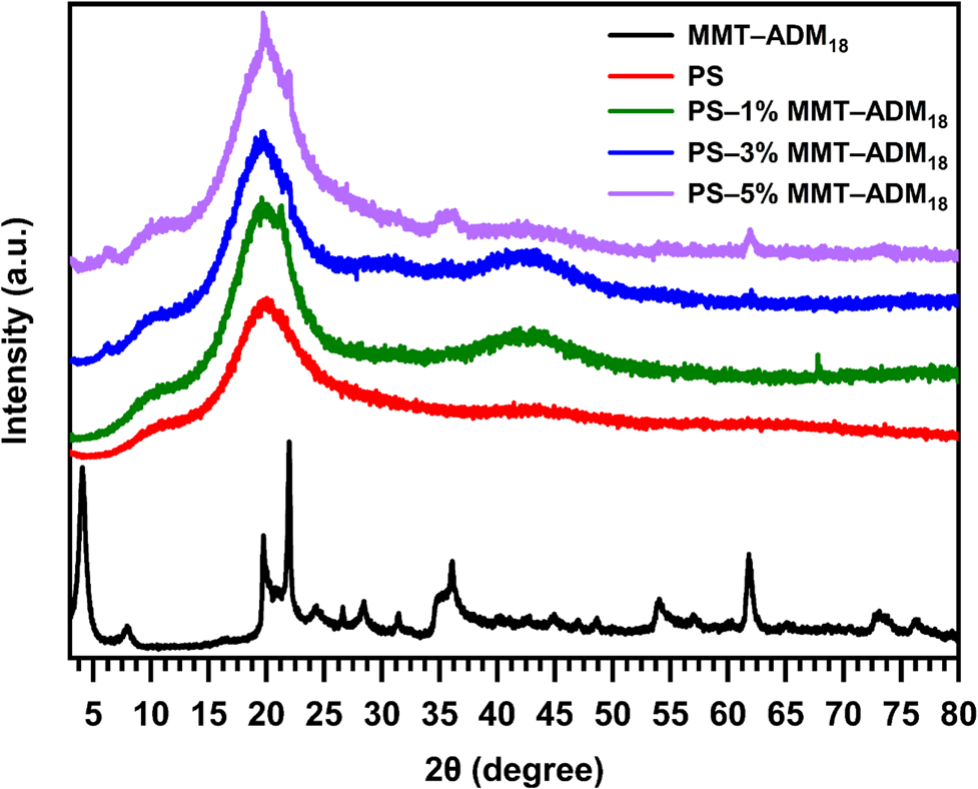
XRD patterns of ADM_18_-modified MMT, and PS/MMT-ADM_18_ nanocomposites with 1, 3, and 5% MMT-ADM_18_.

The diffraction patterns for PS as well as PS nanocomposites with 1, 3, and 5% MMT-ADM_18_ are shown in Fig. 5. PS shows a broad amorphous peak at 2*θ* = 19.5°. After miniemulsion polymerization, the diffraction peak of MMT-ADM_18_, which appeared at 2*θ* = 4° (*d* = 2.18 nm) disappeared in all PS/MMT-ADM_18_ nanocomposite spectra, and the broad peak of PS appeared in all nanocomposite spectra. This indicates that the MMT-ADM_18_ has almost been fully exfoliated in the PS matrix. Table 3 summarizes the calculated exfoliation degree (ED) values from XRD.^44^ The results showed that, as the amount of MMT-ADM_18_ increases, there is a notable decrease in ED values. This indicates that a small amount of MMT-ADM_18_ cannot be exfoliated within the PS matrix. This means that the orderly structure of MMT platelets' becomes disrupted, and layers individually disperse within the polymer matrix. When monomer molecules penetrate the interlamellar space, swelling the MMT, which in turn allows further monomer molecules to enter between layers, thereby facilitating exfoliation.

**Table 3:** Summarizes the estimated exfoliation degree (ED) values of PS/MMT-ADM_18_ nanocomposites

Sample	*d*_001_ (nm)	Exfoliation degree (%)
MMT-ADM_18_	2.18	—
Neat PS	—	—
PS–1% MMT-ADM_18_	None	100
PS–3% MMT-ADM_18_	None	93
PS–5% MMT-ADM_18_	None	83

#### 3.3.3. Morphological structure of nanocomposite materials

**Fig. 6:**
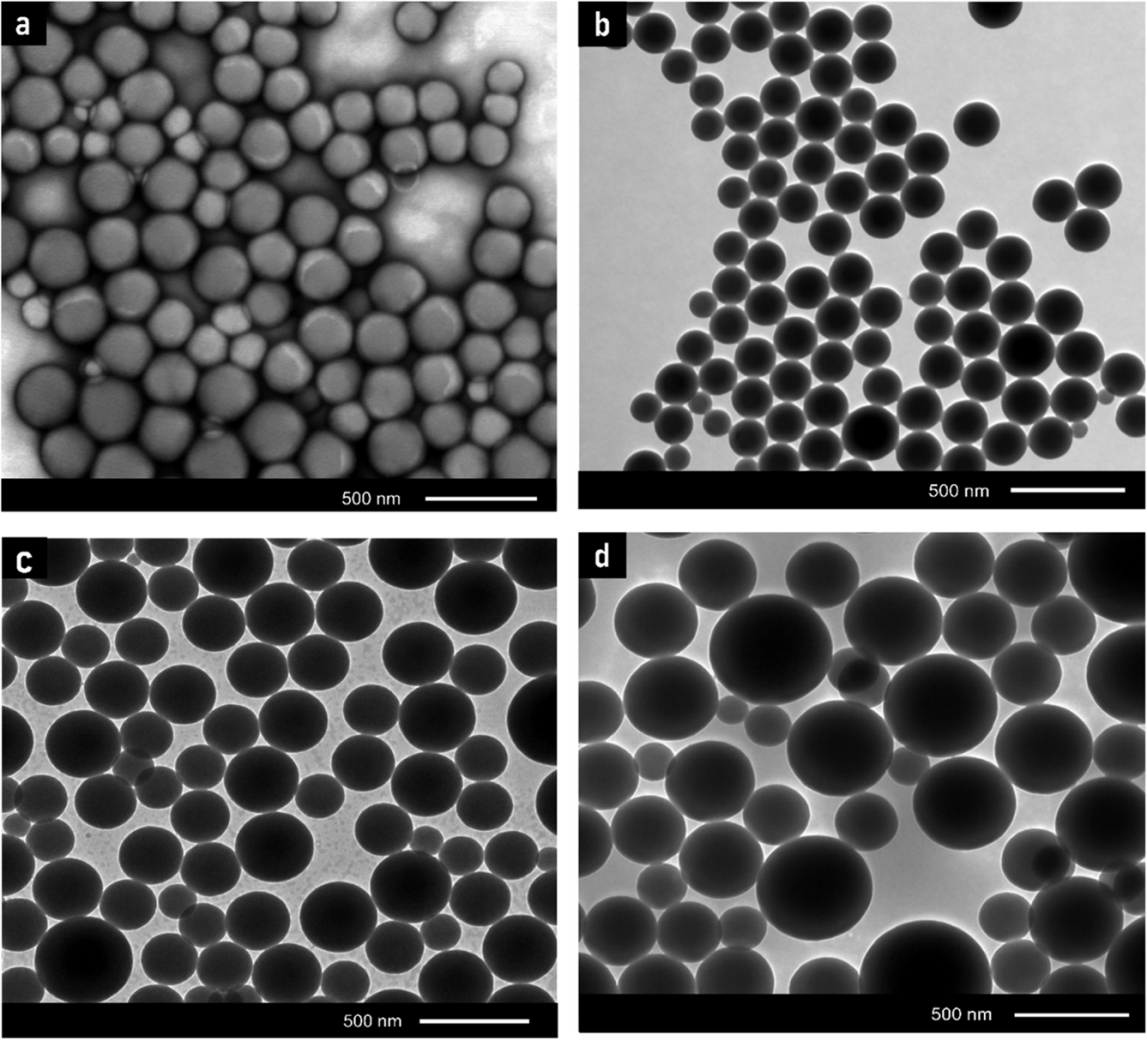
TEM micrographs of the final latex for samples containing (a) 0% MMT-ADM_18_, (b) 1% MMT-ADM_18_, (c) 3% MMT-ADM_18_, and (d) 5% MMT-ADM_18_.

**Fig. 7:**
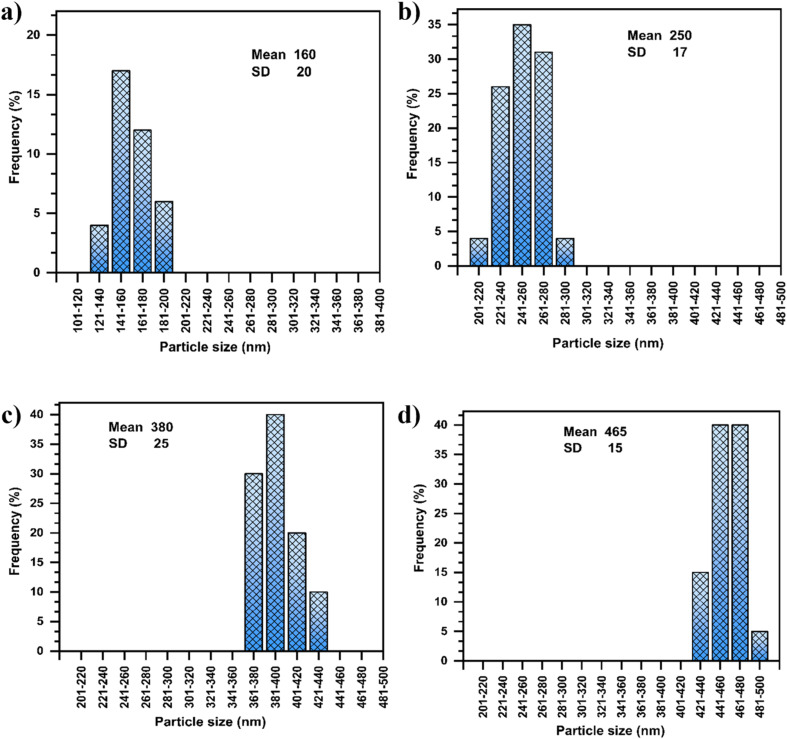
Histograms of particle size distribution derived from TEM images of the latex containing (a) 0% MMT-ADM_18_, (b) 1% MMT-ADM_18_, (c) 3% MMT-ADM_18_, and (d) 5% MMT-ADM_18_.

SEM and TEM were utilized to examine the nanomorphology of PS/MMT-ADM_18_ nanocomposites. Fig. 6 and 7 illustrate TEM images of the final latex as well as the estimated particle size distribution. Fig. 6b displays a TEM image of latex at 1% MMT loading. The latex's particle size is in the range of 250–300 nm (Fig. 7b). Upon a closer look, the image revealed that the particles have a dark inner core surrounded by a light, smooth-contoured PS shell. The dark domain represents MMT platelets (because of MMT's size, the electron's beam cannot pass across the particles, so it displays as dark).^45^ Besides, the latex surface exhibits smooth contours devoid of MMT platelets, indicative of the encapsulation of MMT.

After the MMT content was raised to 3%, the particles seemed larger and exhibited a broad size distribution, as illustrated in Fig. 7c. The resultant latex's average particle size became larger (380 nm) compared to the polymer's average particle size (160 nm) (Fig. 7a). This implies that more MMT platelets are encapsulated in the PS droplets. The particles maintained their distinct morphology, with a large MMT-rich domain with regular contours in the interior and a smooth PS outer layer (Fig. 6c).^46^ After the incorporation of 5% MMT, the latex particle size increased to 465 nm because more MMT platelets were encased in the polymer particles, as shown in Fig. 6d. Large MMT-rich regions in the interior and a smooth-contoured PS shell were observed. Our nanocomposites exhibit much larger particle sizes (2–3 times) compared to the previously reported values.^47^ We also observe that the core's size and dispersion are not homogeneous. There are differences in the number of platelets present in different latex particles. This suggests that when the modified MMT molecule is chemically bonded with the polymer matrix during polymerization, it is possible that the MMT will function as a cross-linking agent since the platelet contains more than one modifying molecule. Therefore, the MMT does not move very easily during polymerization.

**Fig. 8:**
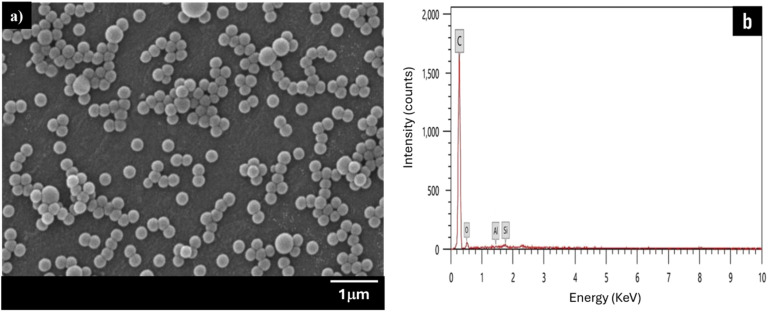
SEM image of (a) final PS nanocomposite latex particles and (b) EDX spectra of the final PS nanocomposite latex particles.

A surface SEM image of latex particles is shown in Fig. 8a, which clearly shows a complete and dispersive spherical morphology. Particles covered with MMT have a rough surface, whereas the noticed latex particles have a smooth surface.^48,49^ Further EDX analysis Fig. 8b discloses that the silicon included in the final latex was not apparent on the latex surface. Therefore, it can be inferred from the previous results that the MMT platelets are within latex particles and not on their surface. Further, SEM and TEM images for the samples containing 1, 3, and 5% of MMT, where the MMT is encapsulated within the polymer particles, are available in Fig. S2.†

#### 3.3.4. Thermal properties

**Fig. 9:**
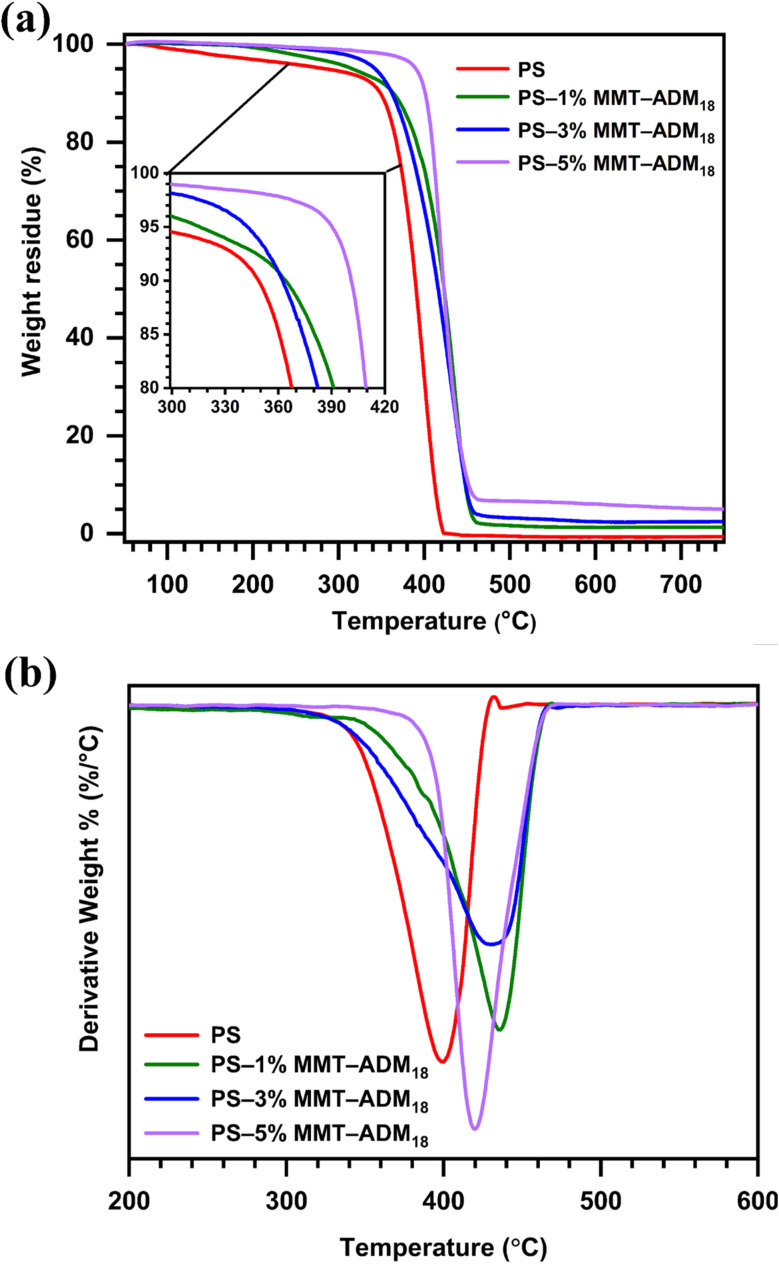
(a) TGA and (b) DTG curves of PS and PS/MMT-ADM_18_ nanocomposites with 1, 3, and 5% MMT-ADM_18_.

The thermal degradation behaviors of PS and PS/MMT-ADM_18_ nanocomposites reveal key thermal stability parameters such as degradation temperature and residual analysis. The TGA curves of PS and PS/MMT-ADM_18_ nanocomposites and their corresponding derivative curves (DTG) are shown in Fig. 9. Table 4 shows the temperatures at 5% degradation (*T*_D5%_), 10% degradation (*T*_D10%_), and 50% degradation (*T*_D50%_), maximum degradation rate (*T*_max_) and char at 800 °C. Fig. 9a and Table 4 show that *T*_D5%_ increased from PS (284 °C) by increasing the amount of MMT-ADM_18_. For example, the *T*_D5%_ increased around 106 °C by the addition of 5% MMT-ADM_18_, compared to the PS sample. The same result was observed with *T*_D10%_. For example, the *T*_D10%_ increased from 349 to 400 °C by adding 5% MMT-ADM_18_. From DTG curves (Fig. 9b), as the amount of MMT-ADM_18_ increases, the temperature at the maximum degradation rate increases. For example, *T*_max_ was increased to 435 °C when 1% MMT-ADM_18_ was added. When the MMT-ADM_18_ amount increased to 5%, the *T*_max_ was increased by 27 °C. The results show that the onset degradation temperatures are higher than those previously reported in the literature.^4,47,50^ The reason behind this noticeable rise in decomposition temperature has been ascribed to either the ability of layered silicate to impede the decomposition products from diffusing or the MMT layers' resistance to heat transmission to PS as a result of their heat capacity. The thermal behavior of the PS/MMT-ADM_18_ nanocomposite was not solely influenced by the amount of MMT but also by the dispersion state, extent of exfoliation, and interplay between the MMT-ADM_18_ and matrix.^6,51^

**Table 4:** Thermal analysis data of PS/MMT-ADM_18_ nanocomposites^a^

Sample	*T*_D5%_	*T*_D10%_	*T*_D50%_	*T*_max_	Char (%)
Neat PS	284	349	390	395	0
PS–1% MMT-ADM_18_	315	364	422	435	1.2
PS–3% MMT-ADM_18_	342	362	417	430	2.7
PS–5% MMT-ADM_18_	390	400	430	422	4.5

a The temperatures at 5% degradation (*T*_D5%_), 10% degradation (*T*_D10%_), and 50% degradation (*T*_D50%_), maximum degradation rate (*T*_max_), and the char yield at 800 °C.

#### 3.3.5. Monomer conversion and latex stability

**Fig. 10:**
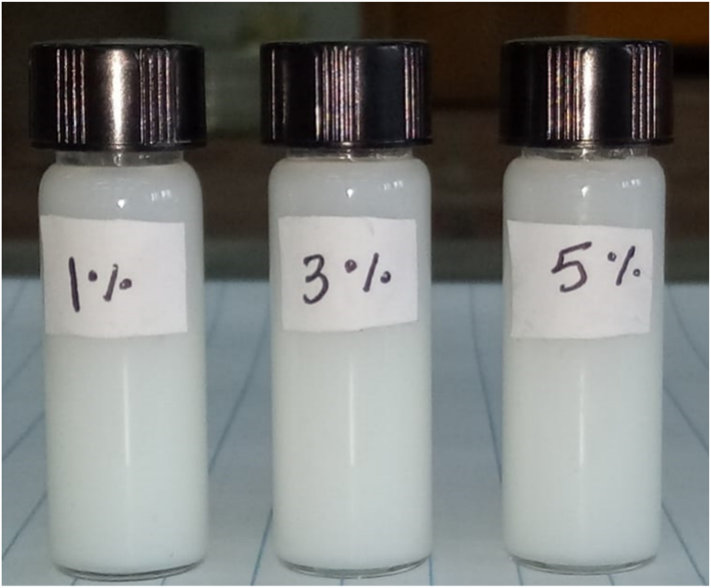
Colloidal stability of final latex containing different amounts of MMT-ADM_18_ over 3 months.

The conversion of monomer with or without MMT-ADM_18_ was determined gravimetrically. The monomer conversion of neat latex reached 96%. As the MMT-ADM_18_ loading increased, a notable decrease in the conversion degree from 92% to 80% was observed. This decrease can be attributed to the contribution of the MMT content, as modified MMT is well known for the increase in viscosity, which hinders the mobility of monomers and disrupts the growth of polymer chains. The covalent anchor between the organic part, which is ionically bonded to MMT, and polystyrene decreases the propagation rate and slows the growth of polymer chains, which in turn leads to an increased termination rate. As a consequence, the conversion rate is reduced. After the polymerization process, the latex was filtered to remove the coagulum. As illustrated in Table 5, loading 1% of MMT yielded a 3% coagulum with an inorganic content of 5%, as determined by calcination. In contrast, increasing MMT loading to 5% resulted in increasing coagulum to 9% with inorganic content of 14%. With varying ratios of MMT-ADM_18_, the final latex demonstrated remarkable stability over three months stored at room temperature (25 °C), as shown in Fig. 10. This can be attributed to the electrostatic stabilization provided by ionic surfactant (SDS), which plays a key role in preventing polymer particle collision and coagulation.

**Table 5:** Summary of final conversion and coagulum percentage for miniemulsion polymerization of PS and its nanocomposites

Sample	Clay content (%)	Conversion^a^ (%)	Coagulum (%)	Inorganic content in coagulum^b^ (%)
Neat PS	—	96	—	—
PS–1% MMT-ADM_18_	1	92	3	5
PS–3% MMT-ADM_18_	3	86	6.6	8
PS–5% MMT-ADM_18_	5	80	9	14

a Determined gravimetrically and.

b Estimated by calcination.

## 4. Conclusion

In this work, using miniemulsion polymerization, modified MMT platelets were encapsulated inside the PS particles. Pre-modification of MMT is key to ensure optimal monomer compatibility and to avoid platelet migration to the polymer/water interface during polymerization. The resultant emulsions exhibited high stability up to a three-month period at 25 °C. The XRD data revealed exfoliation of the final nanocomposites' structure due to MMT-ADM_18_ platelets' uniform distribution throughout the PS matrix. TEM images showed that the average particle size of MMT-ADM_18_ platelets encapsulated inside the PS particles ranged from 250 to 465 nm. The SEM image of the final latex also confirmed encapsulation, as clay platelets were not observed on the surface of the nanocomposite particles. All PS/MMT-ADM_18_ nanocomposites showed a higher degradation temperature than a neat polymer, indicating better thermal stability. The MMT encapsulation within polymer particles may result in materials with niche applications, particularly in fields like paints, coatings, and biomedical fields.

## Data availability

The data supporting this article has been included as part of the ESI.†

## Conflicts of interest

There are no conflicts to declare.

## Supplementary Material

RA-015-D4RA08943J-s001
