# Supplementary material for: Nanocomposites of organo-montmorillonite/polystyrene latex particles via free radical miniemulsion polymerization
Source: RSC Adv. 2025 Feb 18;15(7):5537–46. doi: 10.1039/d4ra08943j (PMC11833771; doi:10.1039/d4ra08943j)
Supplement: RA-015-D4RA08943J-s001 [file RA-015-D4RA08943J-s001.pdf]

This journal is © The Royal Society of Chemistry 2025

# Supporting Information

**For**

# Nanocomposites of organo-montmorillonite/polystyrene latex particles via free radical miniemulsion polymerization

Ahmed Akelah, Ahmed Rehab, Hisham Harhash, Mohamed A Abdelwahab*, Hamada S. A. Mandour

Polymer Research Group, Chemistry Department, Faculty of Science, Tanta University, Tanta, 31527, Egypt.

*Corresponding author: mohamed.abdelwahab@science.tanta.edu.eg

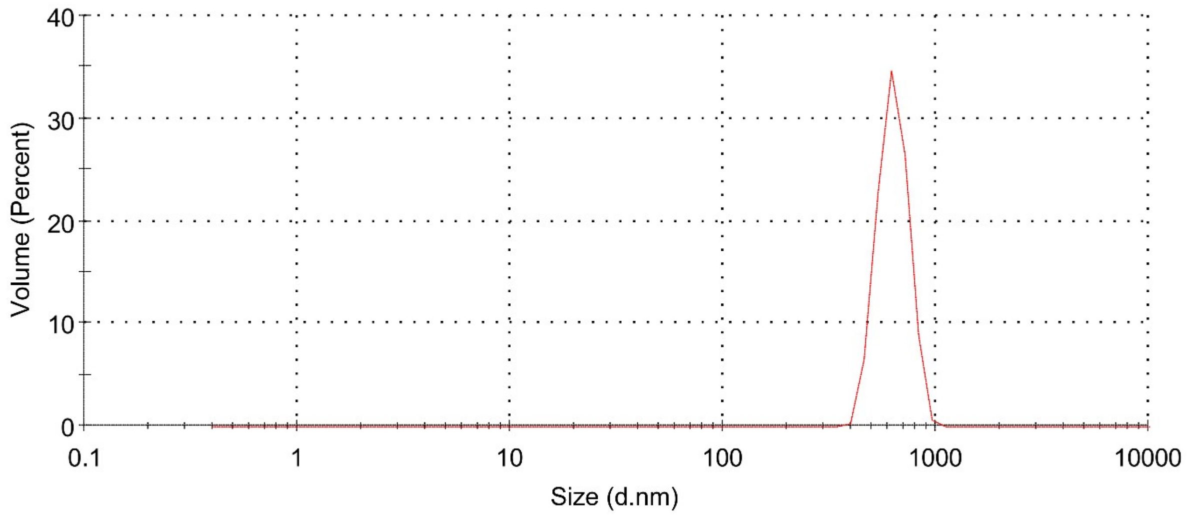


**Figure S1:** DLS plot of MMT-$ADM_{18}$

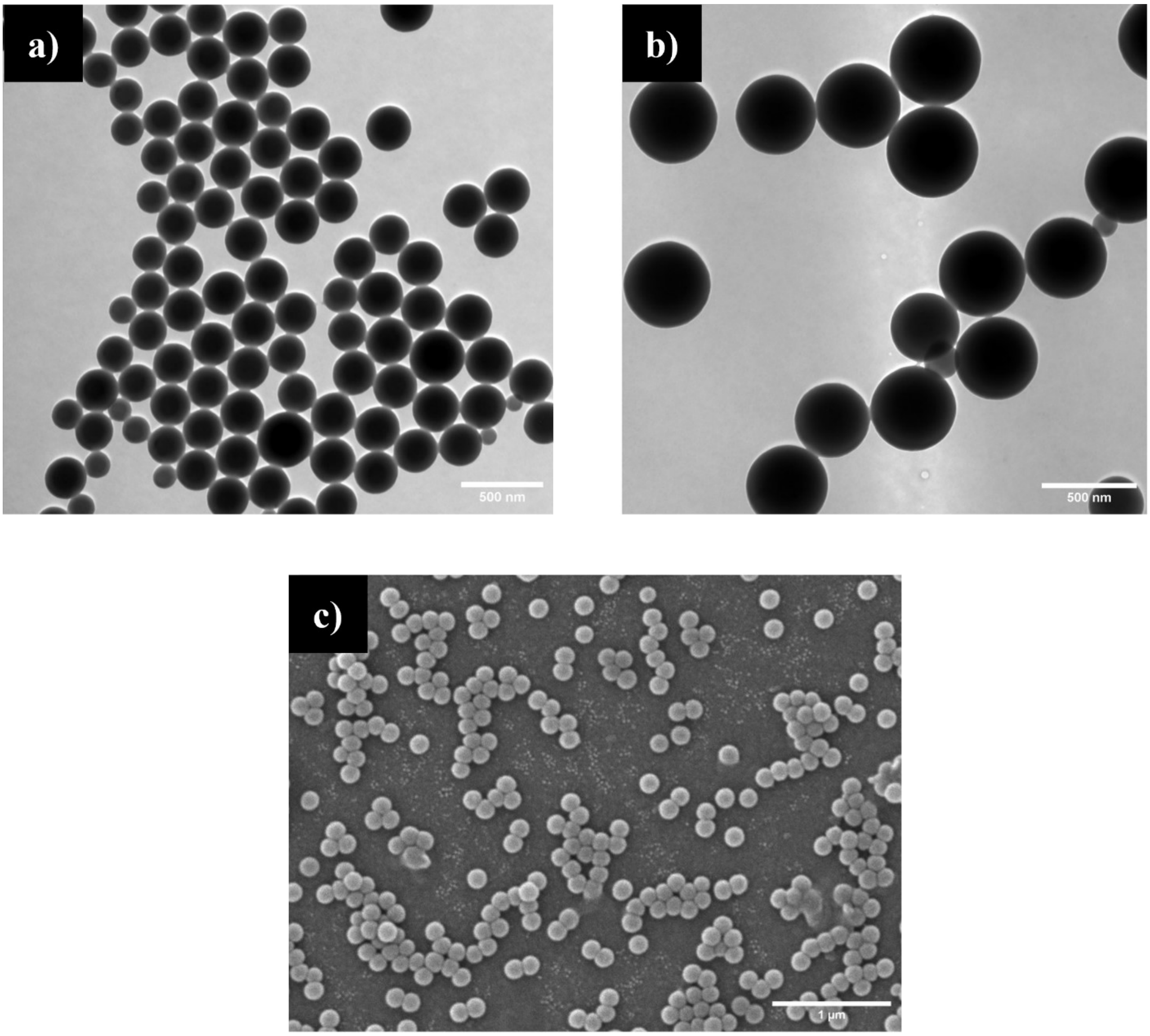


**Figure S2:** TEM images of the final latex for samples with (a) 1% MMT-$ADM_{18}$, (b) 3% MMT-$ADM_{18}$, and (c) SEM image of final latex with 5% MMT-$ADM_{18}$.
